# Association of eNOS Polymorphisms with Anterior Chamber Depth in Han Chinese: Jiangsu Eye Study

**DOI:** 10.1155/2014/164104

**Published:** 2014-02-12

**Authors:** Haihong Shi, Rongrong Zhu, Nan Hu, Jian Shi, Junfang Zhang, Mei Yang, Linjuan Jiang, Hong Jiang, Huaijin Guan

**Affiliations:** Eye Institute, Affiliated Hospital of Nantong University, 20 Xisi Road, Nantong, Jiangsu 226001, China

## Abstract

Recently, a study reported that single nucleotide polymorphisms (SNP) in endothelial nitric oxide synthase (eNOS) were associated with primary angle closure glaucoma (PACG) in Australian cohort. In this study, we aimed to investigate whether those eNOS SNPs are associated with primary angle closure (PAC) or ocular biometric characteristics such as axial length (AL), anterior chamber depth (ACD), and diopter of spherical power (DS) in Han Chinese. The samples consisted of 232 PAC subjects and 306 controls collected from a population-based prevalence survey conducted in Funing County of Jiangsu, China. The rs3793342 and rs11771443 in eNOS were genotyped by TaqMan-MGB probe using the RT-PCR system. Our data did not identify any association of the eNOS SNPs with PAC. However, the analysis on the quantitative traits of ocular biometrics showed that the ACD of rs11771443 AA and GA carriers is significantly deeper than that of rs11771443 GG carriers (*P* = 0.0025), even though the AL and DS are not associated with rs11771443 genotypes. Rs3793342 was not associated with any biometric parameters including ACD, AL and DS. In summary, our data indicates that eNOS rs11771443 is associated with ACD and its role in the pathogenesis of PACG warranted further study.

## 1. Introduction

Glaucoma is the second leading cause of blindness worldwide. Clinically, primary glaucoma presents two major subtypes: primary open-angle glaucoma (POAG) and primary angle closure glaucoma (PACG). The classification relies mainly on the anterior segment anatomy, particularly that of the anterior chamber angle. PACG is characterized by obstruction of aqueous fluid drainage through the trabecular meshwork from the anterior chamber of the eye. The anterior chamber depth (ACD) is a main factor affecting the drainage of aqueous humor. It has been reported that Asian populations are at higher risk of developing PACG than other ethnic groups [1].

Eyes with PACG usually display characteristic anatomical features such as a shorter corneal diameter, a steeper corneal curvature, a shallower anterior chamber, a thicker and more anteriorly positioned lens, and a shortened eyeball, often accompanied by hyperopic refraction error [2]. The risk factors for developing PACG include age, family history, and being female [3]. First-degree relatives were found to have a 6- to 9-fold increased risk of developing PACG [4]. Siblings of Chinese patients with primary angle closure (PAC) or PACG have almost a 50% probability of having narrow angles and are more than 7 times more likely to have narrow angles than the general population [5]. Ethnic differences are also associated with PACG. There is also a higher prevalence among Inuits and Asians compared to Caucasians, suggesting a genetic predisposition for the disorder [6].

Because the ocular anatomic features are predisposing factors for PACG, genes involved in regulation of axial length and structural remodeling of connective tissues may contribute to development of PACG. Some genes related to eye development or tissue remodeling including membrane frizzled-related protein (MFRP) [7, 8], extracellular matrix metalloprotease-9 (MMP-9) [9–11], and methylenetetrahydrofolate reductase (MTHFR) [12] have been reported to be associated with PACG.

Endothelial nitric oxide synthase (eNOS) is a nitric oxide synthase that generates nitric oxide (NO) in blood vessels and is involved with regulating vascular tone by inhibiting smooth muscle contraction and platelet aggregation [13]. Studies also found that eNOS is a pressure-dependent regulator of intraocular pressure and a well-known predisposition gene for POAG [14, 15]. eNOS was recently reported to be associated with PACG assumed by regulating the expression of extracellular matrix metalloproteases [16, 17]. A sequence variation in the intron 4 of eNOS was associated with both POAG and PACG in the Pakistani population [16]. A C-T haplotype established by eNOSrs3793342 and eNOSrs11771443 may be a genetic marker for POAG but not for PACG in the Han Chinese population [18]. Recently, eNOSrs3793342 was reported to be associated with PACG in the Australian population but not in the Nepal population [17]. In short, the relationship of common variations in eNOS and PACG was inconsistent in different populations and the mechanism of eNOS in PACG pathogenesis is unclear. We hypothesized that eNOS might contribute to PACG by influencing ocular anatomical features. Considering that both PACG and POAG are characterized by apoptotic cell death of the retinal ganglion cells in the optic disc or retinal nerve fiber, we attempted to focus on PAC instead of PACG for its possible association with eNOS variants. PAC is the earlier stage of PACG and shares the same anatomical features. We also sought to investigate whether the SNPs of these loci are associated with ocular biometry.

## 2. Methods

### 2.1. Subjects

The study was a part of the Jiangsu Eye Study and was conducted according to the Declaration of Helsinki and approved by the Ethics Committee of the Affiliated Hospital of Nantong University. Each participant was fully informed of the purpose and procedures involved in the study and signed the informed consent form. The general demographic information of the participants is listed in Table 1. All participants were recruited from a population-based prevalence survey on eye diseases using a cluster random sampling strategy in Funing County of Jiangsu, China. Of the 6032 people screened, 232 people with PAC and 306 controls were enrolled in the study. PAC subjects and controls were matched in groups for sex and age and were ethnically homogenous. The participants were unrelated and self-identified Han Chinese. There was no difference between the control group and the PAC group in gender, age, or systemic disease distribution.

All study participants were residents of Funing County of Jiangsu, China, aged 50 years and above. Each participant received a thorough ophthalmic examination, included best-corrected visual acuity, anterior segment photography, Goldmann applanation tonometry, fundus examination, optic disc photography, visual field, objective refraction, and subjective refraction. The depth of the peripheral anterior chamber was determined using Van Herick technique [19]. The subjects with a peripheral chamber depth less than one-third of corneal thickness were invited for gonioscope, A-scan ultrasonography, and ultrasound biomicroscopy (UBM, SW-3200S, SUOER, China) examinations. UBM examinations were conducted in light and dark conditions in eight positions. The detailed protocol for gonioscopy and UBM was reported previously by Barkana et al. [20]. ACD and axial length (AL) were measured 3 times by A-scan to obtain mean values, and mean values of binoculus were used for statistical analyses.

PAC was defined according to the International Society of Geographical and Epidemiologic Ophthalmology (ISGEO) classification by Foster et al. [21]; it includes the following conditions: (1) either eye has the presence of an occluded angle (at least 180 degrees of closed angle in which the trabecular meshwork is not visible on gonioscopy or iris apposition to the trabecular meshwork is more than 180 degrees on UBM); (2) at least one of the following features was detected: peripheral anterior synechiae; intraocular pressure >21 mmHg; excessive pigment deposition on the superior trabecular meshwork; iris whorling; history of symptoms; or intraocular pressure elevated ≥8 mmHg after UBM examination in dark conditions; (3) no signs of secondary angle closure were present; (4) no signs of glaucomatous optic neuropathy and peripheral visual loss were present; (5) no previous ocular surgery or laser therapy was present. The clinical features of the PAC subjects are listed in Table 2.

The criteria for enrollment of the control group were (1) peripheral chamber depth more than one-third of corneal thickness; (2) intraocular pressure less than 21 mmHg; (3) normal optic nerve heads with cup-to-cup ratio less than 0.5; (4) normal visual field; (5) no family history of glaucoma; (6) no ophthalmic diseases except slight cataract; and (7) refractive error of less than three diopters.

### 2.2. SNP Genotyping

Genomic DNA was extracted from the peripheral blood of each individual using the Qiagen Blood DNA Mini Kit (Qiagen, Valencia, CA) according to the manufacturer's instructions and stored at −20°C.

The samples were genotyped for rs3793342 (in intron 4) and rs11771443 (in 5′-UTR region) by TaqMan genotyping assay (Applied Biosystems, Foster City, CA, USA) using the real-time PCR 7500 system (Applied Biosystems, Foster City, CA, USA). PCRs were performed in a total volume of 10 *μ*L containing 1 *μ*L (10 ng) DNA, 5 *μ*L TaqMan Universal Master Mixture, and 0.20 *μ*L TaqMan SNP Genotyping probes (40x). Amplification was carried out with an initial denaturation at 95°C for 5 min, followed by 40 cycles of denaturation at 95°C for 30 s, and annealing at 60°C for 30 s.

### 2.3. Statistical Analysis

Statistical analysis was performed with SPSS version 15.0 software. Differences in age and gender between PAC subjects and controls were assessed using *t*-test and Chi-Square test, respectively. Hardy-Weinberg equilibrium was tested using Chi-Square test. To analyze the association of these two SNPs with PAC and controls, the frequency of genotypes and alleles was evaluated using Chi-Square test. *P* values < 0.05 were considered statistically significant. Logistic regression analysis was performed to calculate the odds ratio (OR) value and the 95% confidence interval (95% CI) and to adjust the confounding effects of age and gender. Three genetic models were analyzed: the additive model defined as minor allele homozygotes versus heterozygotes versus common allele homozygotes, the dominant model as heterozygotes plus minor allele homozygotes versus common allele homozygotes, and the recessive model as minor allele homozygotes versus common allele homozygotes plus heterozygotes. The association of these two SNPs with AL, ACD, and spherical power (DS) was also assessed under the additive genetic model, dominant model using *F*-test, and *t*-test, respectively.

## 3. Results

The call rates of the SNPs genotyped were 100% and the call accuracy was 100% in randomly selected 10% of samples. Both SNPs conformed to Hardy-Weinberg equilibrium (*P* > 0.05) in the PAC group and in the control group.

The two SNPs did not show differences in the distribution of allele frequency (Table 3) and genotypes (Table 4) between the cases and controls.

The ACD of rs11771443 AA and GA carriers is significantly deeper than that of rs11771443 GG carriers (*P* = 0.0025 and *P* = 0.0005 for the additive model and dominant model, resp.). The AL and DS are not associated with rs11771443. Rs3793342 was not significantly associated with biometric parameters including ACD, AL, and DS (Table 5).

## 4. Discussion

This study, to the best of our knowledge, is the first population-based study to investigate the association of rs11771443 and rs3793342 with PAC and PAC relevant biometric parameters such as ACD, AL, and DS in a Han Chinese population. The design of a population-based study can minimize sample selection bias often present in hospital-based case-control study. Our results show that the variations of both SNPs were not associated with PAC. However, the variation of rs11771443 was associated with deeper ACD that is an anatomical feature against PACG. We are not aware of any publications describing the association between rs11771443 and ACD.

eNOS gene is a stress-regulating gene and its expression is triggered when organisms are exposed to stress, hypoxia, or injury. Under these unfavorable conditions, increased NO was produced in tissues to protect themselves against stress [22]. Normal NO level regulates blood flow to the tissues constantly. Low NO level may impair blood flow and related to neurodegenerative disorders, such as optic neuropathy [23]. Studies have found a decrease of nitric oxide in the plasma and aqueous humour of glaucoma patients, and a weak association of nonsynonymous SNP with glaucoma patient with a history of migraine [24]. Nevertheless, an abundance of NO has been found in the optic nerve head vessels of primary glaucoma patients, supporting that optic neuropathy in glaucoma may be related to eNOS overexpression [25]. Awadalla et al. demonstrated that eNOSrs3793342 was associated with PACG and suggested dysregulation of the NO system in this multifactorial optic neuropathy disease [17]. We excluded patients with optic nerve neuropathy from this study to verify the relationship between these SNPs and ocular anatomic features. Taking into account the result that the variation of rs11771443 was associated with deeper ACD, we appraise that the influence of eNOS on PACG may be owing to another mechanism.

The eNOS gene has been shown to play an important role in controlling the activity of matrix metalloproteinases [26]. Dysregulation of the NO system may downregulate MMP9 expression, which has been shown to be associated with PACG [11]. Awadalla et al. postulated that the downregulation in MMP9 activity causes deficiency in extracellular matrix remodeling during eye development and thus leads to hyperopic refractive error and shorter axial length [27]. However, although Awadalla et al. found that eNOSrs3793342 [28] and MMP9rs17576 [27] were associated with PACG, they did not investigate the relationship between these SNPs and AL or refractive status. Interestingly, MMP9rs17576 was also found to be associated with susceptibility to PACG in a Taiwanese population, but there were no differences in AL between the genotypes [11]. Similarly, Cong et al. found that MMP9rs2250889 was associated with PACG in Southern China, but the patients in their study have regular AL and no obvious microphthalmia [10]. In our present study, two SNPs in eNOS were not associated with either PAC or AL and DS. The possible mechanism in which MMP9 and eNOS might contribute to PACG needs to be further studied.

Nathanson and Mckee demonstrated an extensive NO-containing cells in the human ciliary muscle (CM) and outflow pathway [29]. Production of NO in CM could cause a relaxation of the muscle that would counteract the contractile effect of the neuronally released acetylcholine. Contraction of the CM, induced by cholinergic agonists such as pilocarpine, caused forward movement of lens that resulted in shallower ACD [30, 31]. In our present study, we found that the variation of rs11771443 was associated with deeper ACD but not associated with AL and DS. We speculate that the variation of rs11771443 might increase NO production in anterior segment endothelia, result in relaxation of ciliary muscle, and thus increase the depth of anterior chamber. A Singapore study reported that lens vault and posterior corneal arc length were responsible for approximately 75% of the variation of ACD, while axial length was a poor determinant of ACD [32].

In summary, our data indicates that eNOS rs11771443 is associated with ACD and its role in the pathogenesis of PACG warranted further study.

## Figures and Tables

**Table 1:** Demographics of study participants.

Demographic features	Control *n* (%)	PAC *n* (%)	*P*
Female	248 (81.05)	191 (82.33)	0.70
Male	58 (18.95)	41 (17.67)	
Mean age (year) ± SD	65.08 ± 7.53	64.84 ± 8.59	0.74
Age range	50–85	50–83	
Hypertension	66 (19.64)	46 (19.83)	0.69
Diabetes	24 (7.36)	20 (8.62)	0.76
Cardiovascular	10 (3.27)	4 (1.72)	0.41

**Table 2:** Clinical features of PAC subjects.

	Right eye (Mean ± SD)	Left eye (Mean ± SD)	Mean of both eyes (Mean ± SD)
Axial length (mm)	22.17 ± 0.83	22.17 ± 0.82	22.17 ± 0.83
ACD (mm)	2.49 ± 0.29	2.45 ± 0.30	2.47 ± 0.29
Refractive (diopter)	0.53 ± 1.85	0.68 ± 1.87	0.58 ± 1.84
Tonometry (mmHg)	15.18 ± 4.31	15.78 ± 4.46	15.52 ± 4.39

**Table 3:** Allele frequency of SNPs in control and PAC subjects.

SNP	Allele distribution minor/major (minor %)		*P*	OR (95% CI)
	Control	PAC		
eNOS rs3793342 (G/A)	58/554 (9.48)	34/430 (7.33)	0.212	0.76 (0.49–1.17)
eNOS rs11771443 (G/A)	244/368 (39.87)	201/263 (43.32)	0.255	1.15 (0.90–1.47)

All HWE *P* values > 0.05.

**Table 4:** Genotype frequency of SNPs in control and PAC subjects.

SNP	Genotype distribution *n* (%)			General *P* value	Dominant *p*/OR (95% CI)	Recessive *p*/OR (95% CI)
		Control	PAC			
eNOS rs3793342 (G/A)	GG	252 (82.4)	200 (86.2)	0.508	0.23/0.75 (0.46–1.20)	0.63/0.66 (0.12–3.62)
	GA	50 (16.3)	30 (12.9)			
	AA	4 (1.3)	2 (0.9)			
eNOS rs11771443 (G/A)	GG	110 (35.9)	75 (32.3)	0.513	0.38/1.17 (0.82–1.69)	0.32/1.26 (0.80–1.97)
	GA	148 (48.4)	113 (48.7)			
	AA	48 (15.7)	44 (19.0)			

**Table 5:** The relationship of biometric parameters with genotypes of rs3793342, rs11771443 in PAC group.

	Genotype	AL (mm) (mean ± SD)	ACD (mm) (mean ± SD)	Refractives (D) (mean ± SD)
eNOS rs3793342	GG	22.16 ± 0.73	2.46 ± 0.22	0.70 ± 1.47
	GA	22.10 ± 0.81	2.41 ± 0.26	0.76 ± 1.83
	AA	22.29 ± 0.33	2.55 ± 0.09	0.69 ± 1.50
	GA + AA	22.11 ± 0.14	2.42 ± 0.46	0.75 ± 1.79
*p*_*a*_/*p*_*d*_		0.891/0.741	0.444/0.33	0.980/0.848
eNOS rs11771443	GG	22.07 ± 0.77	2.38 ± 0.20	0.42 ± 1.17
	GA	22.22 ± 0.70	2.50 ± 0.25	0.54 ± 1.30
	AA	22.11 ± 0.78	2.49 ± 0.19	0.39 ± 1.25
	GA + AA	22.19 ± 0.72	2.49 ± 0.23	0.50 ± 1.29
*p*_*a*_/*p*_*d*_		0.355/0.245	0.0025/0.0005	0.471/0.475

*p*_*a*_: additive model; *p*_*d*_: dominant model.
